# Sequence-dependence of Cy3 and Cy5 dyes in 3ʹ terminally-labeled single-stranded DNA

**DOI:** 10.1038/s41598-022-19069-9

**Published:** 2022-08-31

**Authors:** Tadija Kekić, Jory Lietard

**Affiliations:** Institute of Inorganic Chemistry, Faculty of Chemistry, University of Vienna, Vienna, Austria

**Keywords:** DNA nanotechnology, Nanobiotechnology, Analytical chemistry, Photochemistry

## Abstract

Fluorescence is an ideal tool to see and manipulate nucleic acids, and engage in their rich and complex biophysical properties. Labeling is the preferred approach to track and quantify fluorescence with nucleic acids and cyanine dyes are emblematic in this context. The fluorescent properties of cyanine dyes are known to be sequence-dependent, with purines in the immediate vicinity increasing the fluorescence intensity of Cy3 and Cy5 dyes, and the ability of nucleobases to modulate the photophysical properties of common fluorophores may influence fluorescence measurements in critical assays such as FISH, qPCR or high-throughput sequencing. In this paper, we comprehensively map the sequence-dependence of Cy3 and Cy5 dyes in 3ʹ-fluorescently labeled single-stranded DNA by preparing the complete permutation library of the 5 consecutive nucleotides immediately adjacent to the dye, or 1024 sequences. G-rich motifs dominate the high fluorescence range, while C-rich motifs lead to significant quenching, an observation consistent with 5ʹ-labeled systems. We also uncover GCGC patterns in the extreme top range of fluorescence, a feature specific to 3ʹ-Cy3 and Cy5 oligonucleotides. This study represents the final piece in linking nucleotide identity to fluorescence changes for Cy3, Cy5 and fluorescein in all 3ʹ, 5ʹ, single-stranded and double-stranded DNA formats.

## Introduction

Fluorescence labeling of nucleic acids is a ubiquitous method to detect, monitor, quantify and study DNA and RNA, in vitro and in vivo^1–4^. Far more convenient than radiolabeling and far more sensitive than the UV absorbance of nucleobases, fluorescence detection is commonplace for nucleic acid quantification, particularly qPCR, a staple in molecular biology that has become omnipresent ever since the outbreak of the SARS-CoV-2 virus^5^. Of the plethora of available fluorescent tags, cyanine dyes are perhaps the most common due to their high brightness, molar extinction coefficient and chemical stability^6,7^. Labeling is typically carried out at the 5ʹ or 3ʹ end of the oligonucleotide and at times on both extremities for FRET studies on molecular beacons^8,9^. The covalent attachment of fluorescent tags to nucleic acids brings the aromatic fluorophore in close proximity to the terminal nucleobase, < 1 nm, making it possible to foresee electronic interactions between the two^10^. And indeed, the fluorescence of common cyanine—but also xanthene—dyes is known to be sequence-dependent^11^. Nucleobases in close proximity to the dye are able to quench or enhance fluorescence through a mechanism involving the modulation of the energy barrier associated with rotational isomerization^6,10–13^. In particular, the loss of rotational freedom in the polyaromatic structure of fluorescent dyes is thought to be directly correlated with enhanced fluorescence properties and this chemical rigidity can be the product of π-π stacking^14,15^ where purines are expected to play a greater role. The ability of nucleobases to modulate the fluorescence properties of nearby dyes creates a quantitation bias where equimolar solutions of two different sequences as measured by A_260_ absorbance yields inconsistent fluorescence emission values. Other approaches relying on fluorescence detection such as next generation sequencing are likely to be affected by sequence-specific effects as well^16^.

We have extensively studied the sequence-dependence of Cy3, Cy5, fluorescein (FAM) and other cyanine derivatives by synthesizing complex DNA microarrays using photolithography, where all possible permutations of five consecutive nucleotides (4^5^ possible combination, 1024 unique sequences) immediately adjacent to a terminal fluorophore are available^17–20^. Our results have showed that 5′-guanine positively affects the fluorescence intensity of Cy3 and Cy5 but quenches FAM, whereas 5′-pyrimidines yield lower Cy3 and Cy5 fluorescence but tend to enhance FAM intensity. Interestingly however, we found that in the case of FAM, 5′ and 3′ labeling yielded different motifs of sequence-dependence, with the fluorescence of 3′-FAM oligonucleotides being affected by guanines further away from the 3′-end, in some cases up to 4 nucleotides away from the dye. In addition, 5ʹ and 3ʹ FAM labeling yields different ranges of fluorescence intensities across all 1024 permutations. These observations prompted us to investigate the sequence-dependence of Cy3 and Cy5 dye in the context of 3′ terminally labeled single-stranded oligonucleotides, with the same 5-nt permutation scheme yielding 1024 unique combinations. To do so and to be able to perform a terminal labeling of oligonucleotide at the 3ʹ end, the photolithographic synthesis of DNA microarrays is carried out in the 5′ → 3′ direction using a set of 3′-photoprotected 5′-phosphoramidites, a method which was recently shown to be as efficient as conventional 3′ → 5′ nucleic acid synthesis^21^. We find that the sequence-dependence of 3′-Cy3 and 3′-Cy5 dyes is largely similar to their 5′ counterparts, with 3′dG dominating the high fluorescence range and 3′dC-rich sequences up to 70% less fluorescent than 3′dG sequences. But we also note unusually high fluorescence for 3′-GCGC patterns which were absent in the 5ʹ labeled datasets. These results should serve to finalize a comprehensive understanding of the sequence dependence of Cy3 and Cy5 dyes in all possible contexts, from 5ʹ to 3ʹ to single and double-stranded DNA.

## Results and discussion

**Figure 1:**
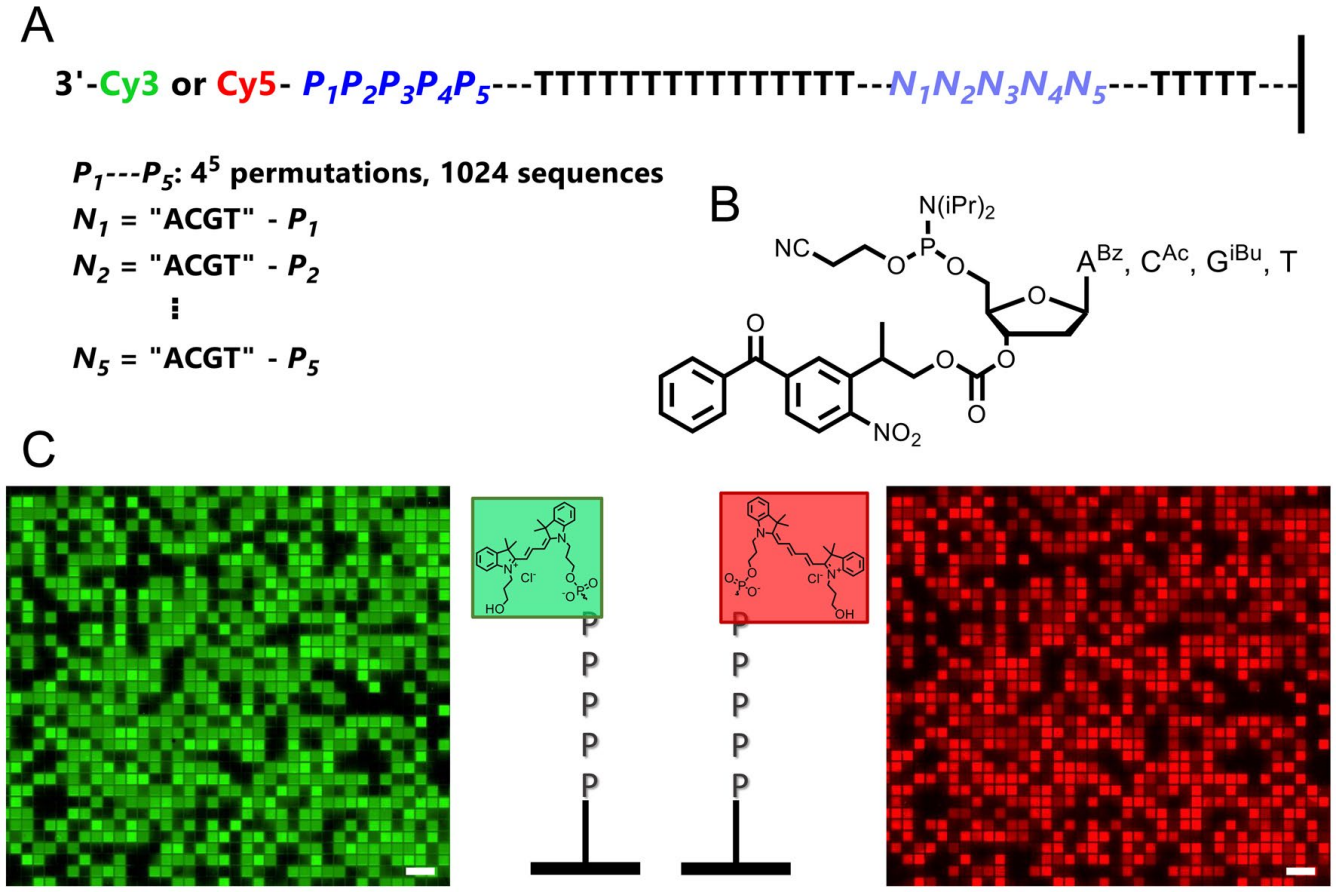
(**A**) Oligonucleotide sequence design for 3′-Cy3 and Cy5 labeled single-stranded DNA. The oligonucleotides consist of a 3′ terminal dye followed by the permutation library (P_1_–P_5_, 1024 combinations) and a T_15_ region. The T_15_ acts as a spacer between the P permutation section and an “adjustment” section composed of 5 trinucleotides N_x_ which are each in the form of 3′-ACGT minus the nucleotide P_x_. A T_5_ linker connects the sequence to the surface of the glass slide. (**B**) Chemical structure of the 3′-BzNPPOC 5′ DNA phosphoramidites used to perform 5′ → 3′ reverse microarray synthesis. (**C**) Excerpts of Cy3 and Cy5 microarray scans (~ 5% of total synthesis area) at 532 and 635 nm, respectively. Single-stranded oligonucleotides are randomly distributed across the surface of the array, separated by non-synthesized space serving as background reference. Scale bar is ~ 200 µm.

We started by studying whether Cy3 and Cy5 attached to the 3′ termini of single-stranded oligonucleotides are differently affected by sequence identity in comparison to 5′ labeled single strands. To do so, we synthesized sequence permutations over five consecutive nucleotides at the 3′ end, which amounts to 4^5^ –or 1024– permutations, using reverse (5′ → 3′) nucleic acid synthesis by microarray photolithography and with a final coupling of Cy3 or Cy5 phosphoramidite (Fig. 1A). Reverse synthesis is very efficient (≥ 98% stepwise efficiency for 3′-BzNPPOC 5′-DNA phosphoramidites, Fig. 1B) and with coupling failures being systematically capped, labeling can only occur on full-length oligonucleotides. To account for the slightly lower coupling efficiency of reverse dG, all oligonucleotide sequences are designed in such a way that they all contain the same amount of G nucleotides, regardless of sequence combination. All sequences being of same length, same distance to the surface and simultaneously synthesized on the same array, the relative fluorescence of each library element can be compared to one another. In addition, the microarray and oligonucleotide sequence design mirrors that of the sequence dependence study on 5ʹ Cy3/Cy5-labeled DNA, allowing for the two datasets to be compared.

**Figure 2:**
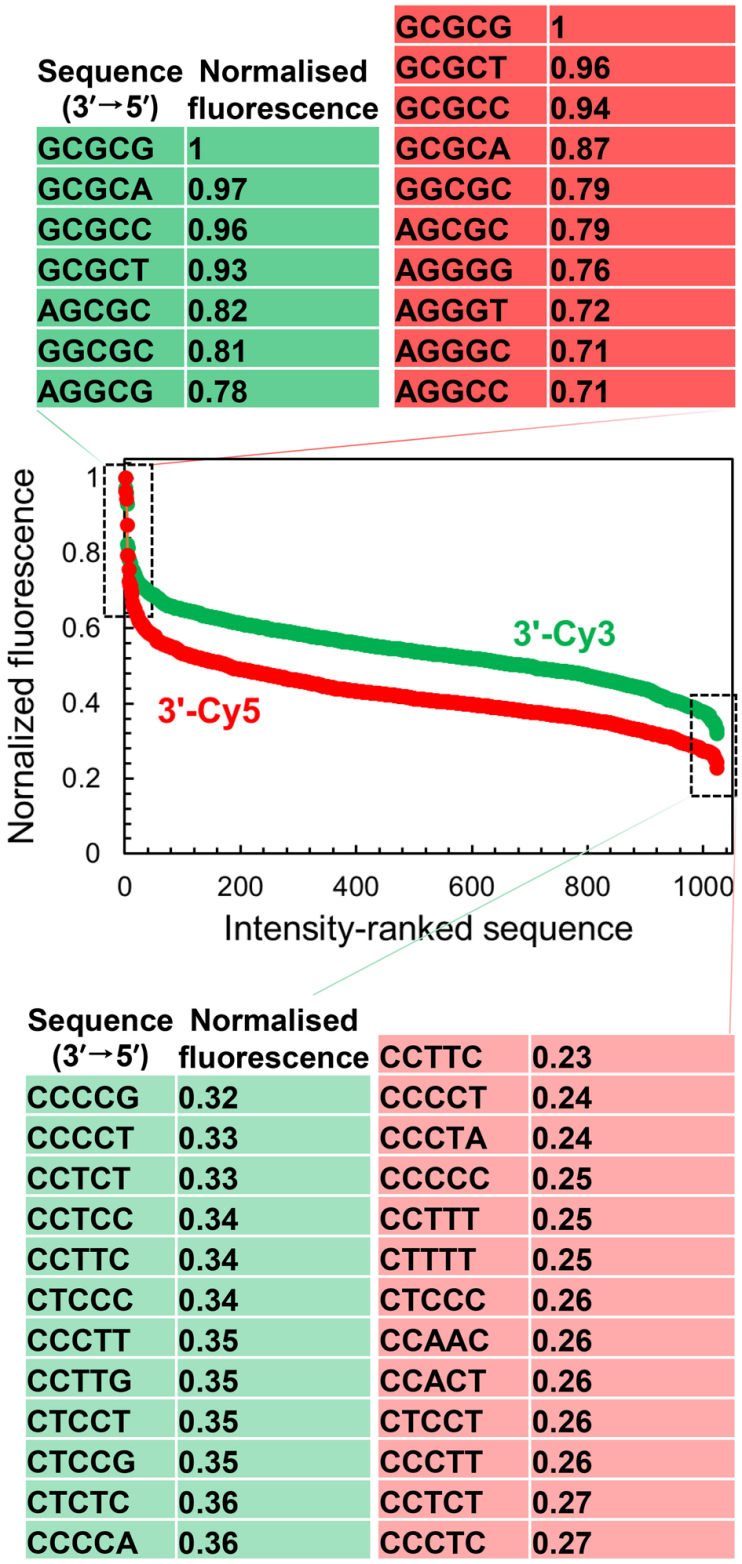
Variations in Cy3 and Cy5 fluorescence intensity as a function of sequence identity in 3ʹ-labeled single-stranded DNA oligonucleotides. The relative fluorescence intensity of Cy3 and Cy5-labeled 5mers was ranked from most to least intense (highest recorded fluorescence and its corresponding 5mer = 1). For Cy3 fluorescence the intensity falls by ≈ 65% and Cy5 by ≈ 73%. A list of the brightest and darkest fluorescent 5mers is shown to the top and bottom part of the panel, respectively.

We indeed find that the fluorescence intensity of Cy3 and Cy5 is heavily affected by the nature of adjacent nucleotides in the context of 3′ terminal labeling (Fig. 1C). We ranked the fluorescence intensity of Cy3 and Cy5-labeled libraries and the distribution adopts a sigmoidal shape in both cases with very large disparities in signal intensity, with a loss of up to 65 and 75% fluorescence relative to the brightest sequence for Cy3 and Cy5, respectively (Fig. 2B). This intensity span is larger than for 5′-Cy3 and Cy5 labeled single-stranded oligonucleotides, where losses of only 50 and 65% were recorded. This larger span of fluorescence intensity in 3ʹ-labeled ssDNA may be explained by the presence of a small number of permutations displaying unusually high brightness in both cyanine cases, as illustrated by the asymmetrical shape of the sigmoidal distribution (Fig. 2). Indeed, the extreme top range of fluorescence intensity is here populated with 5–6 sequence combinations that far outperform the rest of the permutation library.

**Figure 3:**
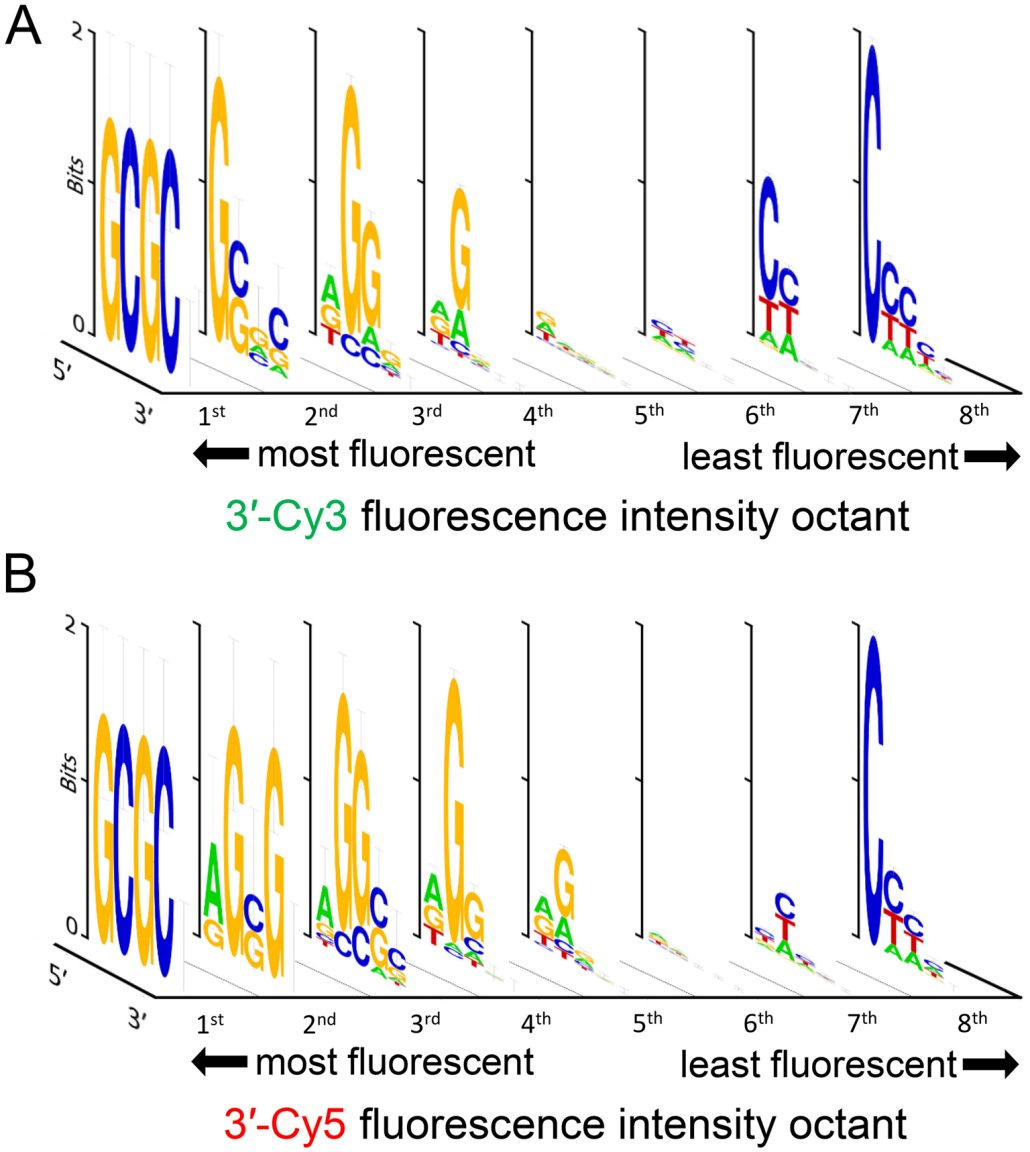
Sequence-dependent variations in the fluorescence intensity of 3′ Cy3- and Cy5-labeled single-stranded oligonucleotides. The intensity range in Figure 2 was divided into 8 equal parts from which consensus sequences were generated and the sequence logos for each octile arranged in descending order of fluorescence intensity (left to right), for 3′-Cy3 (**A**) and 3′-Cy5 labeling (**B**).

These combinations are always of the form 3′-GCGCX and all possible X (A, C, G and T) make up the four brightest Cy3 and Cy5-labeled oligonucleotides (relative fluorescence from 0.87 to 1) and occupying the entire set of sequences in the first octile of Cy3 and Cy5 fluorescence (Fig. 3A,B), hence the very uniform GCGC sequence motif. This observation is particularly surprising in many respects. First, the GCGC pattern is a rare example of a multi-nucleotide motif that influences the fluorescence properties of a nearby dye, when most other systems were found to be primarily affected by the nature of the neighboring base only. Second, this motif introduces two cytidines in the top fluorescence range, when the presence of C is usually synonymous with much lower fluorescence. This can be seen in the low section of Cy3 and Cy5 fluorescence which is associated with C-rich sequences. Third, such a motif was entirely absent from the top-ranking fluorescence in 5ʹ-labeled systems. Finally, this ranking holds true even before deprotection of the DNA microarray, where the nucleobases still carry a base protecting group preventing correct Watson–Crick pairing. The formation of intermolecular G•C base pairs between neighboring strands on the same microarray feature should not occur based on the mean distance between hydroxyl groups at the surface of the glass slide^22^. Similarly, intramolecular hairpin formation with G•C base pairs is unlikely to take place considering the fact that the formation of a loop would be impractical at best. Indeed, with the pattern 3ʹ-GCGCG for instance, the oligonucleotide synthesized on the microarray has the sequence 3ʹ-GCGCGTTTTTTTTTTACTAGTACTAGTACT. Only a single G•C base pair with a 2-nt long G-C loop can occur within the pentamer, and there is no complementarity at all with the adjustment section ACTAGTACTAGTACT. The presence of C nucleotides in high fluorescence range is not unheard of and was observed in 5′-FAM labeled double-stranded DNA^20^, which suggests that loss of rotational motion at the nucleobase level, like after duplex formation, makes it possible to observe fluorescence enhancement with terminal cytidines. Remarkably, the GCGC motif also yields bright fluorescence signals when in the form of 3′-XGCGC, even with X as a terminal pyrimidine. For instance, the 3ʹ-CGCGC combination yields a normalized fluorescence intensity of 0.76 (Cy3) and 0.66 (Cy5), making it the brightest possible combination with a terminal 3ʹ-C, the next instance of any combination in the form of 3ʹ-CXXXX being more than 10% darker than CGCGC.

These XGCGC motifs suggest that the identity of the final nucleobase may not be critical for high fluorescence. Indeed, in the 2nd–4th octiles of Cy3/Cy5 fluorescence (Fig. 3A,B), the identity of the second nucleobase appears to be as important as the terminal base and G nucleotides at the penultimate position occupy a large part of the high Cy3 and Cy5 signal intensity. In the second octile of Cy5 fluorescence (Fig. 3B), the 4th position still shows strong G preference, as strong as the 2nd position, which is likely a consequence of the presence of the GCG motif, a trimmed down version of the GCGC that can still enhance the fluorescence properties of Cy5, though less so for Cy3.

**Figure 4:**
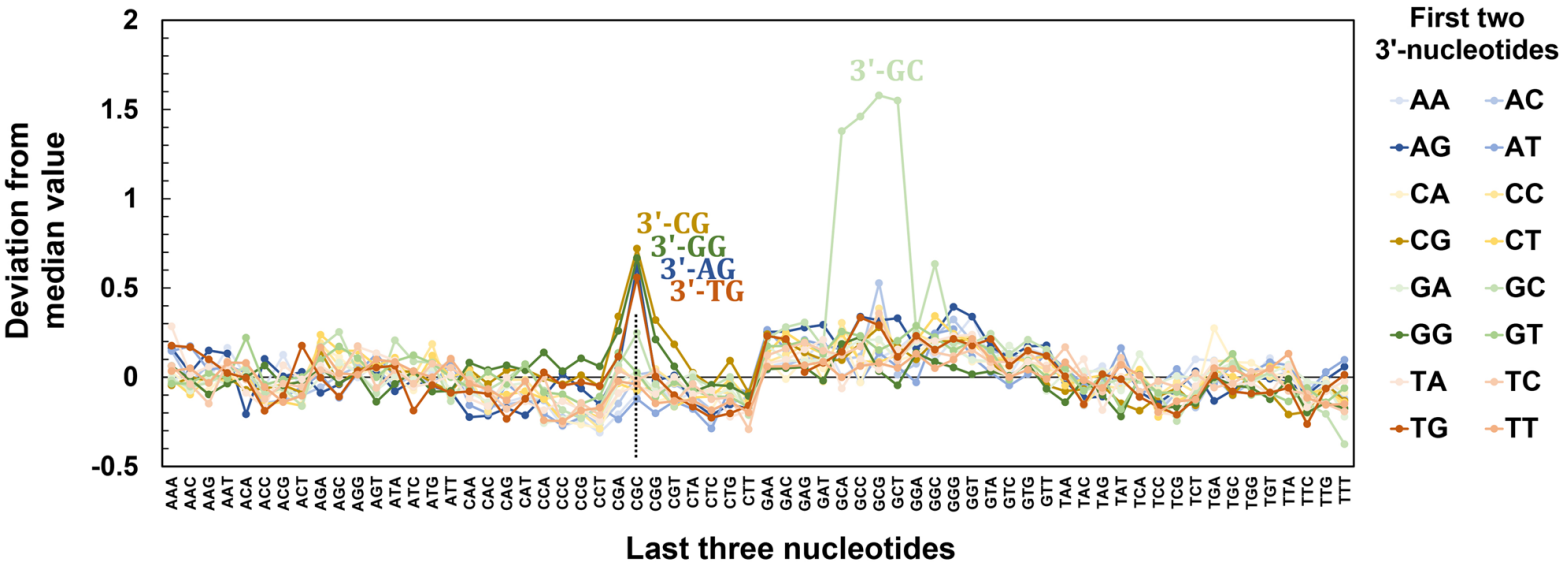
Influence on fluorescence intensity, expressed as deviation from mean fluorescence, of each X_3_X_4_X_5_ trinucleotide combination over each possible X_1_X_2_ dinucleotide in 3ʹ Cy3-labeled X_1_X_2_X_3_X_4_X_5_ pentamers. Mean values are corrected for background fluorescence then standardized across the entire range of Cy3 fluorescence signals. Large deviations are seen for all possible 3ʹ-XGCGC and 3ʹ-GCGXX permutations.

We then looked for patterns further away from the two terminal nucleotide positions. To do so, we studied how, in a system that can be represented in the form 3ʹ-X_1_X_2_X_3_X_4_X_5_, any given X_3_X_4_X_5_ trinucleotide combination affects the fluorescence intensity of each possible X_1_X_2_. We performed a standardization of the fluorescence signals, with a mean value of 0 representing little to no effect. Data above the zero line indicates a positive effect –increase– on fluorescence intensity, and data below the line a negative effect on fluorescence intensity. The results, shown in Fig. 4, further mark the positive influence of GCG systems on fluorescence brightness. Indeed, all 3ʹ-GCGCX combinations tower very significantly above any other XXGCX system. Visibly too, the CGC trinucleotide as X_3_X_4_X_5_ drives the fluorescence signals up only for 3ʹ-XG dinucleotides, a pattern which concatenates into 3ʹ-XGCGC. While this effect could be seen in pure fluorescence rankings (Fig. 2), this particular trinucleotide contrasts with all other in the form CXX which show an overall negative effect on fluorescence intensity. This observation is all the more apparent when comparing to GXX trinucleotides which, in almost all cases, have at least a slight positive effect on fluorescence intensity. Taken together, the data suggests that nucleotide identity three units away from the dye can still be relevant in the context of modulating the fluorescence properties of terminal dyes. Whether the influence of nucleotide X_3_ is due to electronic effects propagating through X_1_ and X_2_, or the result of a direct interaction between 3ʹ-Cy3 and X_3_ is unclear at this point.

Fluorescence enhancement is thought to be directly related to loss of rotational isomerization in cyanine dyes, an effect that would be promoted by stronger stacking systems such as purine rich elements. On the other hand, pyrimidines tracts stack less efficiently, and cytidine less so than thymidine according to free energy measurements^23,24^, which explains why terminal Cs are associated with lower overall fluorescence. Our study focuses on single-stranded DNA, but complementarity between the permutation and “adjustment” sections (Fig. 1A) cannot be excluded. However, we expect hairpin folding to occur for only a few random candidates (Supplementary Table S1) and none of the brightest or darkest sequences can do so. The formation of secondary structures for a small number of sequence permutations should not impact the analysis as it largely stems from pattern search in aggregated data. Indeed, the intensity rankings for deprotected and non-deprotected DNA, where no Watson–Crick structures are possible, overlap fairly well. Our results are consistent with previous observations on the modulation of Cy3/Cy5 fluorescence intensity by neighboring nucleobases, where nucleotide dependency was found to follow the order dG > dA > dT > dC^17,19,25^. The fact that guanines do not quench Cy3 and Cy5 fluorescence is in stark contrast to its effect on other common fluorophores like fluorescein where G-mediated quenching is a known phenomenon^20,26,27^. There, the redox potential of the nucleobase is directly correlated with its ability to quench the fluorescence of the conjugated dye via photoinduced electron transfer, an effect that has recently been described in Cy3-labeled double-stranded DNA^28^. In particular, the fluorescence of Cy3 was found to be dependent on the oxidation potential of the stacking base-pair, with stacking and base-pair formation able to affect (and indeed lower) the oxidation potential of G^28–31^. These effects have not been identified in ssDNA but transient base-stacking interactions in ssDNA cannot be formally excluded and the resulting shifts in redox potential may then contribute to the observed sequence dependence.

The additive effect of multiple Gs or Cs in a row is not immediately apparent, with GGGGG ranking #31 and #21 (0.71 and 0.64 relative intensity) and CCCCC ranking #1009 and #1021 (0.37 and 0.25 relative intensity) for Cy3 and Cy5, respectively. The top-ranking GAAAA motif identified in 5ʹ-Cy3 oligonucleotides here ranks #334 (0.58 relative fluorescence), further illustrating the significant differences between 5ʹ and 3ʹ labeling.

Overall, the dominance of G and C in high and low fluorescence regime is very apparent in both Cy3 and Cy5 cases, as can be seen in Table 1. In the bottom section of both Cy3 and Cy5 fluorescence, the last 83 and 89 pentamers are all in the form 3ʹ-CXXXX and 75% and 98% (Cy3 and Cy5, respectively) of such motifs are under 0.5 relative fluorescence intensity. In contrast, 89% of all 3ʹ-Cy3 GXXXX are above 0.5, but only 25% in the case of 3ʹ-Cy5. G-rich sequences (> 60% of G content per pentamer) are above 0.5 relative fluorescence 99% and 60% of the time.

**Table 1:**
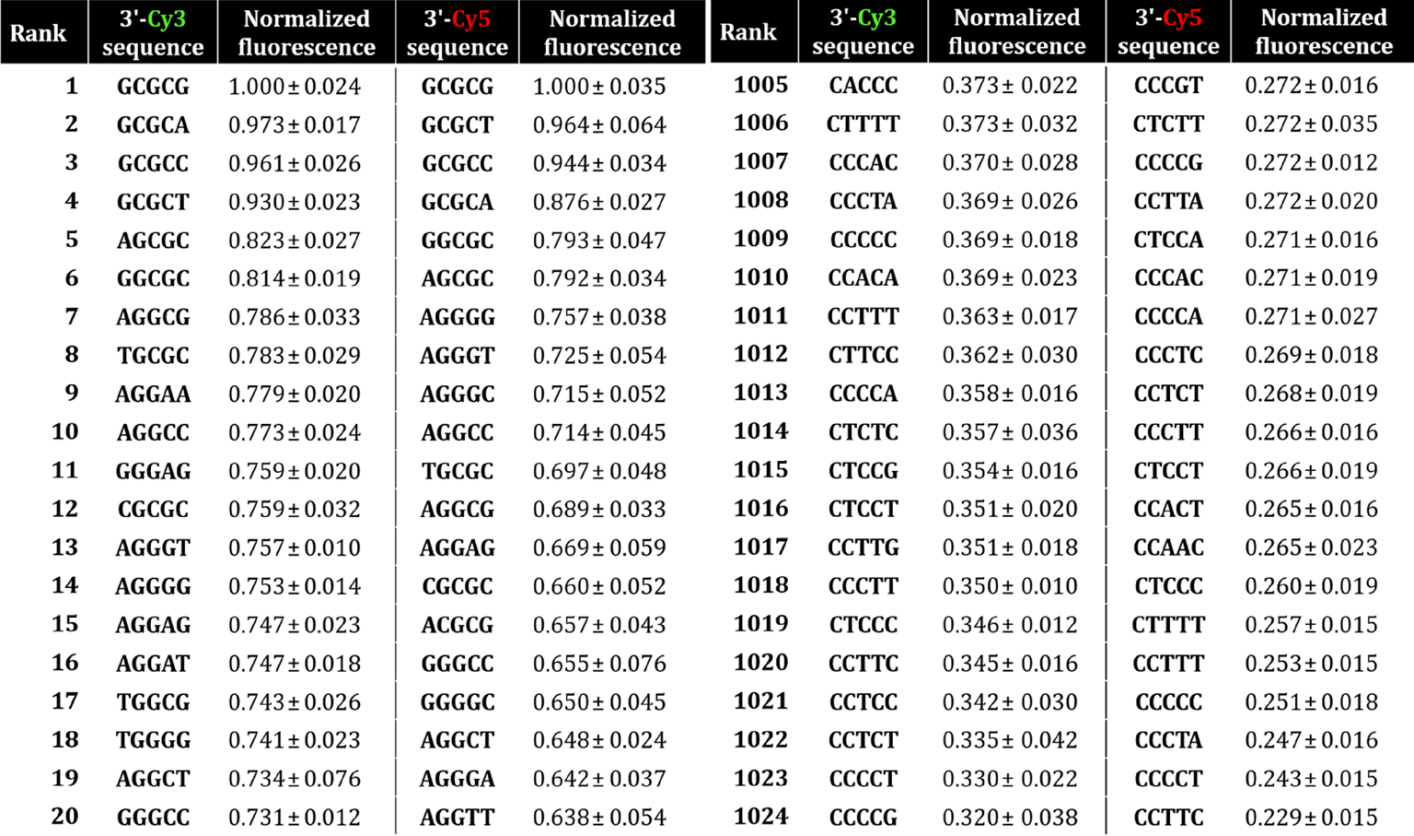
Normalized relative fluorescence intensity of the 20 brightest and darkest pentamers 3ʹ-Cy3 and Cy5 labeled. SEM is standard error of the mean.

## Conclusion

In this study, we looked at the sequence-dependence of 3′ Cy3 and Cy5-labeled single strands by designing a library of oligonucleotides with all possible sequence permutations of the five terminal nucleotides at the 3′ end. The sequence dependence itself is not unlike 5′ labeled Cy3/Cy5 DNA, with G-rich sequences populating high fluorescence signals and, conversely, C-rich sequences quenching Cy3 and Cy5. There are however noticeable differences, with the presence of the 3′-GCGCX motif dominating all permutations (Table 2). We also found that the range of fluorescence change is larger than for the corresponding 5′ labeled sequences, mostly due to the very high fluorescence intensities recorded for all 3′-GCGCX and all 3′-XGCGC combinations. While nucleotide identity at the terminal position is a decisive factor for Cy3 and Cy5 fluorescence intensity, nucleobases further away from the dye can affect fluorescence signals. This is particularly true with dG which can still positively influence Cy3 fluorescence, regardless of the nature of the first two nucleotides at the 3ʹ end. Overall, this study completes the investigation on the sequence-dependence of common nucleic acid fluorophores (Cy3, Cy5 and FAM) in all possible structural contexts, from single to double-strands and from 5ʹ to 3ʹ labeling, which should prove useful in designing carefully calibrated nucleic acid probes carrying fluorophores by taking into account base-mediated fluorescence quenching/enhancement effects. In addition, the large palette of fluorescence intensities for 3ʹ-labeled nucleic acid libraries creates an ideal starting point for mono and polychromic DNA-based painting approaches on microarrays.

**Table 2:** Summary of the most relevant findings and observations in the sequence-dependence of 3ʹ-Cy3 and 3-Cy5 labeled single-stranded DNA.

Observations	3ʹ-Cy3	3ʹ-Cy5
Fluorescent range (norm.)	1–0.35 (65% variation)	1–0.25 (75% variation)
Most fluorescent sequence (#1)	3ʹ-GCGCG	3ʹ-GCGCG
3ʹ-GCGC**X** score	Top 4 most fluorescent	Top 4 most fluorescent
3ʹ-**X**GCGC score	#5, 6, 8, 12	#5, 6, 11, 14
20-most fluorescent sequences	G-rich	G-rich
20-least fluorescent sequences	C-rich	C-rich
3ʹ-CCCC**X** score	#1009, 1013, 1023, 1024	#1007, 1011, 1021
Least fluorescent sequence (#1024)	3ʹ-CCCCG	3ʹ-CCTTC

## Methods

### Sequence design

The design of DNA oligonucleotides is based on a permutation scheme allowing for all possible combinations of 5 consecutive nucleotides immediately adjacent to a fluorescent dye to be synthesized in parallel as microarrays (P_1_–P_5_ in Fig. 1). The total amount of unique pentanucleotides is 4^5^, or 1024 oligonucleotides to be synthesized. To account for potential variability in synthesis efficiency which would produce lower fluorescence signals for poorly-synthesized sequences, the nucleotide content in all DNA sequences is kept constant by adding a subsection composed of five “N” trinucleotides (N_1_ to N_5_) where each N_x_ corresponds to AGCT minus the nucleotide in P_x_. Between the P and N sections, a T_15_ spacer is introduced and the entire oligonucleotide sequence is then synthesized over a T_5_ linker separating the DNA from the surface of the array. At the 3′ end of the oligonucleotide, immediately after the pentanucleotide, a Cy3 or Cy5 dye is attached. Schematically, all 1024 combinations are represented in the form 3′Cy3/Cy5—P_1_P_2_P_3_P_4_P_5_—T_15_—(ACGT-P_1_)—(ACGT-P_2_)—(ACGT-P_3_)—(ACGT-P_4_)—(ACGT-P_5_)—T_5_—glass.

### Microarray synthesis

The process of microarray synthesis by MAS has been extensively described elsewhere^32–38^. Briefly, DNA synthesis takes place on a silane-functionalized glass slide (Schott Nexterion D) and proceeds according to phosphoramidite chemistry. The major change from conventional solid-phase synthesis is the use of a photosensitive BzNPPOC protecting group instead of the acid-labile DMTr, which is removed with 365 nm UV light. To control oligonucleotide elongation and spatial distribution across the surface, patterned UV light is generated by a Digital Micromirror Device (DMD, Texas Instruments) and imaged onto the surface of the slide. DMD mirrors turned ON illuminate the corresponding area of the slide which in turns removes the BzNPPOC protecting group, allowing for nucleoside coupling to take place on photodeprotected areas only. DMD mirrors turned OFF leave the corresponding area on the surface of the array unaffected by UV. Photodeprotection time and efficiency is accelerated by submerging the synthesis area with a 1% solution of imidazole in DMSO. Oligonucleotide synthesis proceeds in the 3′ → 5′ direction using 3′-BzNPPOC protected 5′-DNA phosphoramidites (Orgentis) which were prepared as 30 mM solutions in dry acetonitrile (ACN) and coupled for 60 s in 0.25 M dicyanoimidazole in ACN. Cy3 and Cy5 phosphoramidites (LinkTech) are prepared as 50 mM solutions in ACN and coupled for 2 × 5 min. Before each coupling cycle, the ON areas are exposed for 6 J/cm^2^ in order to reach ~ 99% photodeprotection efficiency. After coupling, unreacted hydroxyl groups are capped by coupling a DMTr-dT phosphoramidite (Sigma-Aldrich) for 60 s. This ensures that the final dye can only be incorporated onto full-length oligonucleotides. Together with high coupling and high photolysis efficiency, terminal 3′ labeling yields a highly uniform oligonucleotide surface density. The delivery of reagents and solvents is accomplished with an Expedite automated DNA synthesizer (PerSeptive Biosystems) which is attached to the reaction chamber encasing the microscope slides.

### Data extraction and analysis

After synthesis, the microarrays are immediately washed in 30 ml ACN for 2 h at r.t. to remove unbound fluorescent phosphoramidite. After drying, the arrays are scanned on a GenePix 4400A or 4100A scanner at 2.5 or 5 µm resolution using the appropriate laser for Cy3 or Cy5 excitation (532 and 635 nm, respectively). The arrays are then transferred into a 1:1 solution of ethylenediamine in ethanol and left to react overnight at r.t. to effect oligonucleotide deprotection. After washing with deionized water (2 × 20 ml), the arrays are dried and scanned again. Data is then extracted from the corresponding scans using NimbleScan software (NimbleGen) and analyzed using Excel. The fluorescence intensity values are calculated as an average of all 10 replicates of each permutation per array to which background fluorescence was subtracted. Background fluorescence corresponds to the fluorescence of non-labeled versions of each oligonucleotide, where the dye binds in a non-specific manner. The reported final fluorescence is an average across at least 3 independent syntheses. Standardization (*z*) of fluorescence signals was performed on background-corrected values (*x*) by applying $z=(x- \mu )/\sigma$ where *µ* is the average fluorescence intensity across all permutations and *σ* the standard deviation of the fluorescence dataset. The consensus sequences were obtained by dividing the range of fluorescence into bins spanning equal intensity, the content of each bin was fed to a sequence logo generator (WebLogo, http://weblogo.berkeley.edu^39^) and the corresponding consensus sequences arranged per bin in decreasing order of fluorescence intensity. Fluorescence intensity data for all permutations is available as part of the supplementary material S1.

## Supplementary Information

Supplementary Information 1.

Supplementary Information 2.

## Data Availability

Supplementary information with the relative fluorescence intensity data for all experimental sequences in spreadsheet format is available for this paper. Microarray scans and raw fluorescence signals are available from the corresponding author upon reasonable request.
