# Supplementary material for: Sequence-dependence of Cy3 and Cy5 dyes in 3ʹ terminally-labeled single-stranded DNA
Source: Sci Rep. 2022 Aug 31;12:14803. doi: 10.1038/s41598-022-19069-9 (PMC9428881; doi:10.1038/s41598-022-19069-9)
Supplement: Supplementary file 1 — Supplementary Information 1. [file 41598_2022_19069_MOESM1_ESM.docx]

**Supplementary Information**

**Sequence-dependence of Cy3 and Cy5 dyes in 3ʹ terminally-labeled single-stranded DNA**

Tadija Kekić and Jory Lietard*

Institute of Inorganic Chemistry, Faculty of Chemistry, University of Vienna, Vienna, Austria

*jory.lietard@univie.ac.at

**Table of Contents**

Table S1. List of permutations with complement sequence within the adjustment section

**Table S1**. List of permutations (P_1_P_2_P_3_P_4_P_5_) with potential for hairpin formation with a 5-bp stem due to self-complementarity to part of the “adjustment” section N_1_N_2_N_3_N_4_N_5_. The complementarity is shown in bold. The size of the loop is not taken into account when considering hairpin stability, only the potential for the entire permuted section to exist as a double-stranded structure.

| **Sequence (3ʹ to 5ʹ)**  (P_1_P_2_P_3_P_4_P_5_TTTTTTTTTN_1_N_2_N_3_N_4_N_5_) |
| --- |
| **AAAAA**TTTTTTTTTT**TTTTT**CGTCGTCGTCGTCGT |
| **GTAGT**TTTTTTTTTTTTTTT**ACTAC**GCGTACTACG |
| **ACTAC**TTTTTTTTTTTTTTTC**GTAGT**ACGCGTAGT |
| **AGTAC**TTTTTTTTTTTTTTTC**GTACT**ACGCGTAGT |
| **CGAGT**TTTTTTTTTTTTTTTAGT**ACTCG**TACTACG |
| **CTAAA**TTTTTTTTTTTT**TTTAG**TACGCGTCGTCGT |
| **CTACG**TTTTTTTTTTTTTTTAGTACG**CGTAG**TACT |
| **CTCGT**TTTTTTTTTTTTTTTAGT**ACGAG**TACTACG |
| **GCGTA**TTTTTTTTTTTTTTTACTAGTAC**TACGC**GT |
| **GTAAA**TTTTTTTTTTTT**TTTAC**TACGCGTCGTCGT |
| **GTACT**TTTTTTTTTTTTTTTACTACGCGT**AGTAC**G |
| **TAAAA**TTTTTTTTTTT**TTTTA**CGCGTCGTCGTCGT |
| **TACGC**TTTTTTTTTTTTTTTAC**GCGTA**GTACTAGT |
| **TACTA**TTTTTTTTTTTTTTTACGCG**TAGTA**CGCGT |
| **TAGTA**TTTTTTTTTTTTTTTACGCG**TACTA**CGCGT |
| **TCGTA**TTTTTTTTTTTTTT**TACGA**GTACTACGCGT |
|  |
